# Deciphering RNA m^6^A regulation in aging: Perspectives on current advances and future directions

**DOI:** 10.1111/acel.13972

**Published:** 2023-08-28

**Authors:** Zeming Wu, Jie Ren, Guang‐Hui Liu

**Affiliations:** ^1^ State Key Laboratory of Membrane Biology, Institute of Zoology Chinese Academy of Sciences Beijing China; ^2^ Institute for Stem Cell and Regeneration Chinese Academy of Sciences Beijing China; ^3^ Beijing Institute for Stem Cell and Regenerative Medicine Beijing China; ^4^ Key Laboratory of RNA Science and Engineering, CAS Key Laboratory of Genomic and Precision Medicine, Beijing Institute of Genomics Chinese Academy of Sciences and China National Center for Bioinformation Beijing China; ^5^ University of Chinese Academy of Sciences Beijing China; ^6^ Sino‐Danish College University of Chinese Academy of Sciences Beijing China; ^7^ Advanced Innovation Center for Human Brain Protection, and National Clinical Research Center for Geriatric Disorders Xuanwu Hospital Capital Medical University Beijing China; ^8^ Aging Translational Medicine Center, International Center for Aging and Cancer, Xuanwu Hospital Capital Medical University Beijing China

**Keywords:** aging, disease, m^6^A, RNA methylation, senescence

## Abstract

N^6^‐methyladenosine (m^6^A) is a dynamic and reversible RNA modification that has emerged as a crucial player in the life cycle of RNA, thus playing a pivotal role in various biological processes. In recent years, the potential involvement of RNA m^6^A modification in aging and age‐related diseases has gained increasing attention, making it a promising target for understanding the molecular mechanisms underlying aging and developing new therapeutic strategies. This Perspective article will summarize the current advances in aging‐related m^6^A regulation, highlighting the most significant findings and their implications for our understanding of cellular senescence and aging, and the potential for targeting RNA m^6^A regulation as a therapeutic strategy. We will also discuss the limitations and challenges in this field and provide insights into future research directions. By providing a comprehensive overview of the current state of the field, this Perspective article aims to facilitate further advances in our understanding of the molecular mechanisms underlying aging and to identify new therapeutic targets for aging‐related diseases.

**Abbreviations**
3'UTR: 3' untranslated region
AD: Alzheimer's disease
AGO2: Argonaute 2
ALKBH5: AlkB homolog 5
Cas9: CRISPR‐associated protein 9
CDKN1A: cyclin‐dependent kinase inhibitor 1A
circRNA: circular RNA
CRISPR: clustered regularly interspaced short palindromic repeats
DGCR8: DiGeorge syndrome critical region 8
DPSCs: dental pulp stem cells
ERVs: endogenous retroviruses
FF: follicular fluid
FOS: Fos proto‐oncogene
FTO: fat mass and obesity‐associated protein
GCs: granulosa cells
GLORI: glyoxal and nitrite‐mediated deamination of unmethylated adenosines
HGPS: Hutchinson‐Gilford progeria syndrome
IGF2BP1: insulin‐like growth factor 2 mRNA‐binding protein 1
IGF2BP2: insulin‐like growth factor 2 mRNA‐binding protein 2
LECs: lens epithelium cells
LINE‐1: long interspersed element‐1
m^6^A: N^6^‐methyladenosine
m^6^A‐SAC‐seq: m^6^A‐selective allyl chemical labeling and sequencing
METTL14: methyltransferase like 14
METTL3: methyltransferase like 3
miRNA: microRNA
MIS12: MIS12 kinetochore complex component
mRNA: messenger RNA
MSCs: mesenchymal stem cells
NF‐κB: nuclear factor‐kappa B
NMDAR1: N‐methyl‐d‐aspartate receptor 1
NPCs: nucleus pulposus cells
NPNT: nephronectin
PC12: rat pheochromocytoma cell line
PD: Parkinson's disease
PLK1: Polo‐like kinase 1
Pth1r: parathyroid hormone receptor 1
SA‐β‐gal: senescence‐associated β‐galactosidase
SIRT1: Sirtuin 1
TNF‐α: tumor necrosis factor‐alpha
WS: Werner syndrome
YTHDF: YTH domain‐containing family protein

## 1 INTRODUCTION

Aging is a complex biological process characterized by the progressive deterioration of physiological functions and is associated with increased susceptibility to various age‐related chronic diseases (Cai, Ji, et al., 2022; Cai, Song, et al., 2022; Kennedy et al., 2014; Ren et al., 2023; Sun, Li, & Kirkland, 2022). Deciphering the molecular mechanism of aging is crucial for developing effective interventions to combat aging‐associated degeneration and promote healthy aging (Cai, Ji, et al., 2022; Cai, Song, et al., 2022; Li, Xiong, et al., 2023; Liu et al., 2022; Wang et al., 2022; Zhang, Qu, et al., 2020). Similar to DNA and protein, RNA can also be decorated with a variety of chemical modifications, leading to a special layer of epigenetic regulation known as the epitranscriptome (McMahon et al., 2021; Zhao et al., 2017). Currently, over 170 types of RNA modifications have been discovered, among which N^6^_‐_methyladenosine (m^6^A) is reported as the most common and conserved modification in eukaryotic mRNA. RNA m^6^A modification is dynamically regulated by “writers” such as METTL3 and METTL14, “erasers” such as FTO and ALKBH5, and “readers” such as YTH family members and IGF2 binding proteins. These regulators function to control splicing, nuclear export, stability, and translation of target RNAs involved in various biological processes (Bai et al., 2023; Deng et al., 2018; Huang et al., 2020; Roignant & Soller, 2017). Recently, there has been increasing evidence for the involvement of m^6^A regulation in cellular senescence and other aging‐related processes (Bao et al., 2023; Casella et al., 2019; Jiapaer et al., 2022; Sun, Cheng, et al., 2022; Wu et al., 2022; Zhang & Xia, 2023). This offers a new perspective on understanding and combating aging and highlights the potential for targeting m^6^A regulation as a therapeutic strategy.

In this Perspective article, we provide an in‐depth overview of recent advances in m^6^A epitranscriptomic regulation during aging. Specifically, we will focus on the roles and mechanisms of m^6^A methyltransferases and demethylases in cellular senescence and tissue aging. We will examine how m^6^A regulation affects key players in cell cycle‐related events and tissue homeostasis. Finally, we will also discuss the current limitations of our understanding of m^6^A epitranscriptomic regulation during aging and highlight potential opportunities for future research.

## 2 m^6^A REGULATION IN CELLULAR SENESCENCE

**FIGURE 1:**
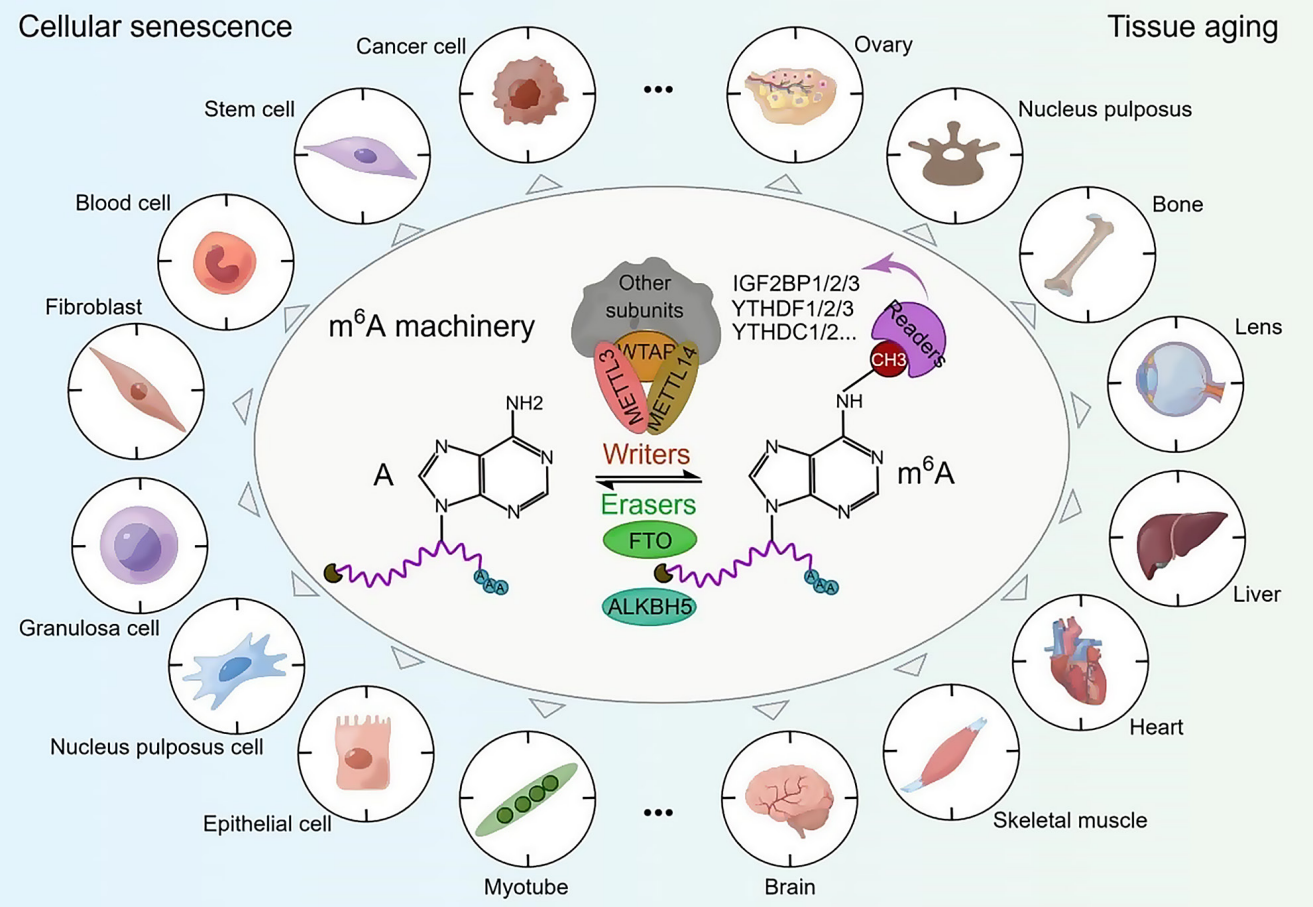
The m^6^A regulatory machinery and its association with senescence or aging in a variety of representative cell and tissue types. *The schematic diagram is prepared by Figdraw*.

Cellular senescence, characterized by cell cycle arrest, is a critical hallmark and driver of tissue degeneration and aging. Emerging evidence has implicated RNA m^6^A regulation in the process of cellular senescence (Figure 1), which may act by altering the levels of cell cycle regulators. For instance, researchers have reported that METTL3/METTL14 catalyzes m^6^A modification in the 3'UTR of *CDKN1A* mRNA, which encodes the cyclin‐dependent kinase inhibitor p21, to promote cell cycle arrest in oxidative stress‐induced senescence of human cancer cells (Abbas & Dutta, 2009). Interestingly, knockdown of METTL3 or METTL14 decreased the protein level of p21 but had no effects on its overall mRNA level, thus demonstrating a role of m^6^A in the enhancement of p21 translation (Q. Li et al., 2017). The tumor suppressor p53, which functions as an upstream factor of p21 to induce cell cycle arrest (Engeland, 2022), is also involved in m^6^A‐associated regulation of cellular senescence. For example, in sulforaphane‐induced senescence of human cancer cells, upregulation of p53 and p21 concomitantly occurred with a reduction in the overall m^6^A abundance, which is also accompanied by alterations in global DNA methylation (Lewinska et al., 2017). This work implies a potential involvement of m^6^A regulation in chemical‐induced cellular senescence as well as a possible interplay between RNA modification and DNA methylation in this process, while the underlying mechanism remains unclear. Similarly, m^6^A‐mediated regulation of other cell cycle‐related factors has also been reported in human stem cell senescence. For example, knockdown of METTL3 led to accelerated senescence of human dental pulp stem cells (DPSCs), as indicated by increased staining of senescence‐associated β‐galactosidase (SA‐β‐gal) and disturbed cell cycle progression. This process was potentially mediated by the upregulation of PLK1, a critical cell cycle regulator, at both the mRNA and protein levels due to decreased m^6^A modification (Luo et al., 2021). Additionally, genetically modified human mesenchymal stem cell (MSC) models have been established to recapitulate two typical premature aging diseases, that is, Hutchinson‐Gilford progeria syndrome (HGPS) and Werner syndrome (WS). In both progeroid MSC models, downregulation of METTL3 was detected along with a global reduction in RNA m^6^A abundance during premature senescence. Moreover, CRISPR/Cas9‐mediated knockout of METTL3 accelerated senescence in wild‐type human MSCs, while overexpression of METTL3 rescued the premature senescence of progeroid MSCs. By profiling the m^6^A modification across the whole transcriptome, MIS12, another cell cycle modulator, whose methylation and expression levels were both diminished in senescent cells, was identified as a key downstream regulator of m^6^A in counteracting human MSC senescence. Further analysis identified that the m^6^A reader IGF2BP2 was involved in the recognition and stabilization of *MIS12* mRNA (Wu et al., 2020). These findings offer novel insights into the epitranscriptomic regulation of human stem cell senescence and provide potential interventions by targeting cell cycle regulators to combat aging‐associated diseases.

RNA m^6^A modification has also been implicated in miRNA processing and subsequent gene expression modulation (Roundtree et al., 2017). Thus, it is worthwhile to investigate whether m^6^A can participate in the regulation of cellular senescence via miRNA‐related mechanisms. Indeed, in physiologically senescent human blood cells, m^6^A epitranscriptomic analysis identified a global decrease in the RNA methylation level when compared to that in young cells. Interestingly, a decrease in the methylation and expression level of *AGO2* mRNA, which encodes a key enzyme in the RNA interference machinery, was observed in both physiologically senescent blood cells and replicatively senescent fibroblasts. Subsequently, researchers demonstrated that METTL3/METTL14‐mediated m^6^A modification promoted the stability of *AGO2* mRNA and the subsequent expression of mature miRNAs. Meanwhile, knockdown of METTL3 or METTL14 accelerated the cellular senescence of fibroblasts (Min et al., 2018). Although this study couples senescence‐associated m^6^A regulation with miRNA processing, future mechanistic studies will be of great significance to address which m^6^A reader stabilizes methylated *AGO2* mRNA and which miRNAs play a dominant role in counteracting senescence. m^6^A‐associated miRNA processing was also reported in human nucleus pulposus cell (NPC) senescence, as knockdown of METTL14 alleviated TNF‐α‐induced cell cycle arrest and senescence of NPCs via inhibiting the m^6^A and expression level of miR‐34a. Mechanistic insights identified an interaction between METTL14 and DGCR8 in promoting the processing of miR‐34a, which targets SIRT1 to accelerate NPC senescence (Zhu et al., 2021). These findings demonstrate the involvement of m^6^A‐mediated miRNA and its target mRNA regulation in cellular senescence. However, it is important to note that this field of research is still evolving, and further studies are needed to fully understand the underlying mechanisms and functional consequences.

Notably, m^6^A methyltransferases or demethylases also appear to regulate cellular senescence in an m^6^A‐independent manner. One example of such regulation is observed in oncogene‐induced senescence of human fibroblasts, where METTL3 and METTL14 facilitate chromatin remodeling via promoter‐enhancer looping, leading to the expression of senescence‐associated secretory phenotype (SASP) genes driven by NF‐κB p65. This regulation was independent of m^6^A modifications (Liu et al., 2021). Another intriguing finding is the interaction between METTL3 and METTL14 with Lamin A in both human and mouse cells. This interaction serves to ensure the correct positioning of nuclear speckles and protects these methyltransferases from proteasome‐mediated degradation (Zhang, Ao, et al., 2020). In this study, downregulation of METTL3 and METTL14 was associated with replicative senescence in normal human fibroblasts or premature senescence in fibroblasts from both HGPS patients and progeroid mice. Conversely, overexpression of METTL14 restored heterochromatin and nuclear organization, effectively alleviating cellular senescence. Although a specific role for m^6^A in this model was not declared in the study, it appears that METTL3 and METTL14 can antagonize cellular senescence independently of m^6^A modifications. Furthermore, FTO has also been implicated in the regulation of cellular senescence without m^6^A involvement. For instance, in human MSCs, depletion of FTO disrupted its interaction with MIS12, leading to a decrease in the level of MIS12 protein, rather than its mRNA form, and an accelerated senescence phenotype. Further evidence shows that FTO interacts with MIS12 and safeguards it from proteasomal degradation, although the detailed mechanism remains to be explored (Zhang et al., 2022). These findings highlight the significant role of m^6^A methyltransferases and demethylases, such as METTL3, METTL14, and FTO, in regulating cellular senescence. The evidence presented here demonstrates that their impact extends beyond traditional m^6^A modifications, emphasizing the need to explore alternative mechanisms through which these enzymes modulate cellular senescence pathways and providing valuable insights into the intricate nature of senescence regulation.

## 3 m^6^A REGULATION IN TISSUE AGING

Either increased or decreased m^6^A modification has been reported in various tissues, contributing to degenerative progression and aging (Figure 1). To unravel the epitranscriptomic mechanisms underlying aging, it is crucial to understand the relationship between m^6^A dysregulation and its effects on different target RNAs, as well as the involvement of upstream regulatory enzymes in specific tissue types. In a study published in 2021, researchers identified downregulated FTO expression and an overall increase in RNA m^6^A modification in human follicular fluid (FF) and granulosa cells (GCs) from aged donors and murine ovaries from old mice (Sun et al., 2021). Shortly afterwards, another study claimed a similar dysregulation of m^6^A modification and FTO expression in GCs derived from aged human ovaries. Using in vitro cultured human ovarian granulosa cell lines, the researchers found that knockdown of FTO accelerated GC senescence, which was attenuated by FOS inhibition, identifying *FOS* mRNA as a key downstream effector of m^6^A. Further analysis revealed that IGF2BP2 was responsible for the stability of m^6^A‐tagged *FOS* mRNA (Jiang et al., 2021). Similarly, patients with intervertebral disc degeneration, a common aging‐associated disorder, exhibited elevated m^6^A modification levels in nucleus pulposus (NP) tissues, which positively correlated with METTL14 and TNF‐α expression (Zhu et al., 2021). Using human NPCs as a cellular model, the authors found that TNF‐α treatment induced upregulation of METTL14 and m^6^A and subsequent premature senescence. This can be reversed by METTL14 knockdown, potentially through a mechanism associated with miRNA regulation, as described earlier in Section 2 (Zhu et al., 2021). Taken together, these studies suggest that increased m^6^A deposition could contribute to aging acceleration in certain tissue types.

On the contrary, reduced m^6^A levels can also promote tissue degeneration or aging in certain contexts. For instance, conditional knockout of *Mettl3* in mice led to reduced m^6^A levels and shortened lifespan, along with obvious degenerative alterations in bone, as characterized by decreased bone mass and increased marrow fat accumulation. Overexpression of Mettl3 prevented estrogen deficiency‐induced osteoporosis, an age‐related skeletal disorder, by facilitating the translation of *Pth1r* mRNA, which encodes a protein critical for bone formation, although the functional m^6^A reader of *Pth1r* mRNA remains uncharacterized (Wu et al., 2018). Additionally, m^6^A decline is associated with human lens aging (Li et al., 2020). For example, a study by Li et al. reported decreased m^6^A abundance in total circRNAs in lens epithelium cells (LECs) from patients with age‐related cataracts. This reduction was potentially due to the upregulated expression of ALKBH5 in LECs. The regulatory network for ALKBH5‐mediated m^6^A upregulation in this process remains to be clarified, but this study provides insights into the mechanism and potential target of ophthalmic degeneration disorders.

Recent studies have illustrated the m^6^A regulatory maps in multi‐tissue aging. Among them, our team profiled the m^6^A landscape in the liver, heart, and skeletal muscle during nonhuman primate aging, revealing a positive correlation between RNA m^6^A modification and gene expression homeostasis across tissues, as well as tissue type‐specific RNA methylation dynamics with aging (Wu et al., 2023). We found that skeletal muscle was most susceptible to m^6^A loss and METTL3 downregulation during aging, which was associated with age‐related phenotypes such as reduced fiber cross‐sectional area, augmented expression of inflammatory factors, decreased protein level of Lamin B1, and elevated apoptosis. Using human pluripotent stem cell‐derived myotubes as a cellular model, we identified METTL3 deficiency as a potential driver of skeletal muscle aging and NPNT as a key downstream factor of m^6^A in maintaining myotube homeostasis. Overexpression of METTL3 counteracted myotube senescence by upregulating NPNT in an m^6^A‐dependent manner, and IGF2BP1 was involved in *NPNT* mRNA stabilization. These findings provide epitranscriptomic insights into skeletal muscle aging and providing potential therapeutic targets to treat sarcopenia, another age‐associated disease commonly seen in the clinic. Previous studies found that METTL3 inhibition resulted in impaired skeletal muscle regeneration in mice (Liang et al., 2021) and that METTL3 knockout in mice led to muscle wasting and abrogated the overload‐induced hypertrophy (Petrosino et al., 2022), supporting our findings. Together, these findings suggest a geroprotective role of METTL3 in promoting skeletal muscle homeostasis and provide promising potential to develop novel interventions for the treatment of age‐related diseases, such as sarcopenia.

Apart from the aforementioned tissues, increasing evidence has pointed to an important role for m^6^A regulation in brain aging and neurodegenerative diseases. For example, Shafik et al. described increased m^6^A modification in brain aging in both mice and humans (Shafik et al., 2021). Unexpectedly, they detected a decrease in the m^6^A level in a mouse model of Alzheimer's disease (AD). Combined with a *Drosophila* AD model expressing human Tau, this study associated the inhibition of METTL3, METTL14, or YTHDF with enhanced Tau toxicity, thus hinting at a potential role of m^6^A in the regulation of AD pathogenesis. However, Castro‐Hernández et al. revealed a substantial reduction in m^6^A‐methylated transcripts in the brains of aged mice and human AD patients (Castro‐Hernández et al., 2023). They also provided evidence at the mechanistic level, which indicated that METTL3‐mediated m^6^A modification was related to the synthesis of synaptic proteins. Inconsistently, yet another study reported a global increase in m^6^A modification in the brains of AD mice, concomitant with the upregulated expression of METTL3 and downregulated expression of FTO (Han et al., 2020). The possible explanations for the inconsistency among these studies deserve further investigation. Altered m^6^A modification has also been involved in another neurodegenerative disease, that is, Parkinson's disease (PD). In a rat model of PD, a global reduction in RNA m^6^A levels was observed in the brain (Chen et al., 2019). Further analysis in 6‐hydroxydopamine‐treated PC12 cells revealed that FTO‐mediated m^6^A removal was associated with the upregulation of NMDAR1 expression, oxidative stress, and Ca^2+^ influx, which may contribute to dopaminergic neuron apoptosis and PD progression.

## 4 CONCLUSION AND PERSPECTIVES

This Perspective article provides an overview of m^6^A regulation in cellular senescence and tissue aging, as well as a variety of age‐related disorders, focusing on the roles of m^6^A methyltransferases and demethylases and the underlying mechanisms. It offers epitranscriptomic insights into the molecular networks of aging and the development of potential therapeutic strategies. This encompasses the involvement of common regulatory pathways in distinct cellular and tissue contexts. Specifically, it has been consistently observed that abnormal expression of cell cycle‐related factors, mediated by m^6^A dysregulation, occurs in various types of human stem cells and cancer cells. This phenomenon may arise due to cell cycle arrest serving as a potentially universal hallmark of cellular senescence (Di Micco et al., 2021; Gorgoulis et al., 2019; López‐Otín et al., 2023; Ogrodnik et al., 2019; Wang et al., 2023), involving the expression control of a range of cell cycle regulators and persisting even across distinct cell types. The article also notes cell‐ or tissue‐specific effects of m^6^A regulation on aging‐related processes. As an example, our recent study reported a noticeable decline in both METTL3 expression and m^6^A modification specifically during the aging of primate skeletal muscles (Wu et al., 2023). Interestingly, this decline was not observed in aged livers or hearts, although both cardiac and skeletal muscles consist of postmitotic and highly differentiated myofibers. Although the underlying roots of such tissue specificity remain elusive, variations in cellular and molecular heterogeneity among different tissues may contribute to m^6^A‐associated aging regulation (He et al., 2020; Tian et al., 2023; Trapp et al., 2021; Yamamoto et al., 2022). Overall, we provide a comprehensive summary of m^6^A regulatory networks in aging at both the cellular and tissue levels, deepening the understanding of complex biological changes associated with senescence or aging. More importantly, the Perspective article also discusses the implications of m^6^A dysregulation in age‐related diseases such as osteoporosis and AD, highlighting the potential for the development of novel therapeutic strategies to ameliorate age‐related pathologies by targeting the m^6^A machinery.

Despite the valuable insights gained from m^6^A research, there are limitations and challenges that need to be addressed. For example, inconsistencies among studies on AD may be due to variations in research contexts and lack of standardized methodologies, highlighting the need for further investigations to ensure reproducibility and reliability in our understanding of m^6^A regulation during aging. Establishing uniform standards for animal or cell model construction, sample collection and processing, as well as guidelines for sequencing and analysis methodologies, may be helpful to address such issues. Looking ahead, there are several promising avenues for future research. First, uncovering more comprehensive and in‐depth mechanisms of m^6^A regulation in cellular senescence, aging or aging‐related diseases will provide valuable insights into the underlying molecular pathways and targets. For example, emerging studies have shown an important association of m^6^A in the regulation of retrotransposable elements including long‐interspersed element‐1 (LINE‐1) and endogenous retroviruses (ERVs), thus being involved in the homeostatic maintenance of stem cells (Chelmicki et al., 2021; Della Valle et al., 2022; Wei et al., 2022; Xu et al., 2021). Considering the critical involvement of LINE‐1 and ERVs in aging (Bi et al., 2020; De Cecco et al., 2019; Gorbunova et al., 2021; Liu, Liu, et al., 2023; Simon et al., 2019; Zhang et al., 2023), it would be very interesting to investigate whether m^6^A could regulate the activity of these retrotransposons in the aging process. Filling this knowledge gap can potentially lead to the novel development of targeted interventions to delay or mitigate age‐related processes. Second, exploring the broader landscape of m^6^A modifications across more tissues, cell types, and species will deepen our understanding of their regulatory functions during aging. This could be achieved through the combined application of advanced sequencing methods that enable high‐resolution profiling of m^6^A modifications at the single‐base or single‐cell level, such as m^6^A‐SAC‐seq (Hu et al., 2022), GLORI (Liu, Sun, et al., 2023), and single‐cell m^6^A‐seq (Li, Wang, et al., 2023; Yao et al., 2023). Finally, translating the findings from preclinical models and cellular systems to the clinic remains a crucial question. Further evaluation is needed to determine whether the mechanistic insights gained from model organisms and cellular models can be easily translated to humans and applied in clinical settings. A systematic research platform combining human stem cells, organoids, nonhuman primates, and clinical samples would be beneficial. Taken together, continued research focusing on addressing the current limitations and challenges in this field holds great potential for our understanding of the epigenetic regulation of aging and the development of novel therapeutics via targeting m^6^A regulatory machines to advance our ability to mitigate age‐related diseases.

## AUTHOR CONTRIBUTIONS

G.H. L. and J.R. conceived the idea. Z.W. drafted the manuscript. All authors shared responsibility for editing both the form and content of this manuscript and approving the final version.

## CONFLICT OF INTEREST STATEMENT

The authors declare no conflicts of interest.
